# Family medicine residents’ skill levels in emergency chest X-ray interpretation

**DOI:** 10.1186/s12875-021-01390-3

**Published:** 2021-02-17

**Authors:** Malak Al Shammari, Ali Hassan, Nouf AlShamlan, Sarah Alotaibi, Manar Bamashmoos, Amani Hakami, Abdullatif Althunyan, Shymaa Basager, Sameerah Motabgani, Sawsan Aljubran, Hind S. Alsaif

**Affiliations:** 1 Department of Family and Community Medicine, Imam Abdulrahman Bin Faisal University, Al-Khobar, Saudi Arabia; 2 Department of Radiology, Salmaniya Medical Complex, Manama, Bahrain; 3 Department of Radiology, King Fahd Hospital of the University, Imam Abdulrahman Bin Faisal University, Al-Khobar, Saudi Arabia

**Keywords:** Chest X-ray, Diagnostic accuracy, Emergency medicine, Family medicine, Residency program

## Abstract

**Background:**

Family medicine physicians may encounter a wide variety of conditions, including acute and urgent cases. Considering the limited access to diagnostic investigations in primary care practice, chest X-ray remains the imaging modality of choice. The current study assessed the competency of family medicine residents in the interpretation of chest X-rays for emergency conditions and to compare it with that of diagnostic radiology residents, general practitioners, and medical interns.

**Methods:**

An online survey was distributed to 600 physicians, including family medicine residents, medical interns, general practitioners, and diagnostic radiology residents. The study included some background information such as gender, years in practice, training type, interest in pulmonary medicine and diagnostic radiology, and having adequate training on the interpretation of chest X-rays. The survey had 10 chest X-ray cases with brief clinical information. Participants were asked to choose the most likely diagnosis and to rate their degree of confidence in the interpretation of the chest X-ray for each case.

**Results:**

The survey was completed by 205 physicians (response rate = 34.2%). The overall diagnostic accuracy was 63.1% with a significant difference between family medicine and radiology residents (58.0% vs. 90.5%; *P* < 0.001). The COVID-19 pneumonia (85.4%) and pneumoperitoneum (80.5%) cases had the highest diagnostic accuracy scores. There was a significant correlation between the diagnostic confidence and accuracy (*r*_*s*_ = 0.39; *P* < 0.001). Multivariable regression analysis revealed that being diagnostic radiology residents (odds ratio [*OR*]: 13.0; 95% confidence interval [*CI*]: 2.5–67.7) and having higher diagnostic confidence (*OR*: 2.2; 95% *CI*: 1.3–3.8) were the only independent predictors of achieving high diagnostic accuracy.

**Conclusion:**

The competency of family medicine residents in the interpretation of chest X-ray for emergency conditions was far from optimal. The introduction of radiology training courses on emergency conditions seems imperative. Alternatively, the use of tele-radiology in primary healthcare centers should be considered.

**Supplementary Information:**

The online version contains supplementary material available at 10.1186/s12875-021-01390-3.

## Background

Family medicine is a unique specialty because the scope of practice is not limited to a particular age, gender, or organ system. As the first point of contact within the healthcare system, family medicine physicians may encounter a broad spectrum of medical conditions, with different complexities, ranging from minor complaints to life-threatening emergencies. The provision of emergency care for acute and urgent conditions is a pivotal role of family medicine practice. It is estimated that emergency cases constitute approximately 5% of all cases in primary healthcare centers [1]. The primary care centers may not be adequately equipped for proper management of emergency cases [2]. Physicians in primary healthcare centers depend largely on their skills in history taking and physical examination to establish a diagnosis considering limited access to other assessment tools [3].

The chest X-ray is a crucial diagnostic tool because it is widely available, relatively inexpensive, and has a low dose of radiation exposure. The chest X-ray remains the starting point among imaging modalities for the diagnosis and management of cardiac and pulmonary conditions. Several studies have demonstrated the fundamental role of chest X-ray in clinical decision-making [4–6]. For instance, a prospective cohort study involving 78 general practitioners revealed that the proposed management was altered in 60% of patients who underwent a chest X-ray with a reduction in referrals, increased satisfaction of patients, and improved prescribing [4].

Most primary healthcare centers may not have access to radiologists for the interpretation of radiographs. Family medicine physicians, therefore, must rely on their own radiologic interpretive skills. Several studies have investigated the agreement rate between family medicine physicians and radiologists and found the discordance rates may be as high as 58.1% [7, 8]. While the majority of this discordance may not cause substantial changes in patient care [8, 9], misinterpretation of chest X-rays of some emergencies could result in serious outcomes [10].

According to the curriculum of the Saudi Board for Family Medicine Program, family medicine residents are expected to master the necessary knowledge and skills for requesting and interpreting chest X-ray images; however, little is known about the competency of family medicine residents in the interpretation of chest X-rays for emergency conditions. It should be pointed out that diagnostic radiology is not given adequate consideration in the undergraduate medical education and graduates may not feel sufficiently prepared for accurate radiological interpretation in medical practice [11].

Therefore, it would be of particular interest to assess the competency of family medicine residents in the interpretation of chest X-rays and to compare diagnostic accuracy and confidence with that of diagnostic radiology residents and physicians who have not undergone a postgraduate training program, including medical interns and general practitioners.

## Methods

### Study design

After Ethical Review Board approval, a survey-based cross-sectional study was conducted to investigate the competency of family medicine residents in the interpretation of chest X-rays for acute and emergency conditions. The survey was designed using the QuestionPro (Seattle, WA, USA) platform. The survey was anonymous and without a time limit.

The survey was accompanied by a cover letter describing the purpose of the study and the voluntary nature of participation. Participants were encouraged to contact the research investigator with any queries pertaining to the study using the provided contact information. Participants were also informed that they could receive the correct answers after the study is completed upon request.

### Study subjects

The study focused primarily on the family medicine physicians practicing in hospitals in the Eastern Province of Saudi Arabia. Diagnostic radiology residents and physicians who have not undergone postgraduate training were included to allow for comparison of results. Most participants were recruited from the Imam Abdulrahman Bin Faisal University, the affiliated institution of the research investigators, since their contact information was readily available.

### Survey distribution

The survey was distributed via e-mail and WhatsApp (Facebook, Menlo Park, CA, USA) messages to 600 physicians. Each invitation had a unique link that could not be used more than once so that the survey was not compromised by duplicate responses. A reminder e-mail was sent after 3 days. Participants were able to access the survey on their mobile phones, tablets, or computers. The survey commenced on July 1, 2020 and was open for 14 days.

### Survey content

The questionnaire began with background questions, including gender, years in practice, type of training, interest in pulmonary medicine and diagnostic radiology, having completed an elective course in diagnostic radiology, and perceived to have adequate training in interpreting chest X-ray images. The survey included a series of 10 cases that started with a brief clinical vignette and a chest X-ray image followed by a multiple-choice question asking for the most likely diagnosis. For each case, participants were also asked to rate their degree of confidence using a 5-point scale. The survey included an optional free-text field for participants to provide feedback or comments (Supplementary File 1).

### Selection of chest X-rays

The chest X-ray images were selected from Radiopaedia, an international radiology educational web resource, after obtaining permission. The cases were selected to represent common emergency conditions that are encountered in medical practice. The chest x-ray images were independently interpreted by two fellowship-trained general body radiologists who were not involved in selecting the images and had complete agreement with the diagnoses of all cases. A pilot study was conducted with 10 physicians, with different levels of experience, to assess the clarity of cases and the time required to complete the survey. The participants reported that the survey was quite long and required > 30 min to complete. Hence, the number of cases was reduced from 15 to 10 cases with the aim of improving the anticipated response rate [12].

### Statistical analysis

Data were compiled using the QuestionPro platform and analyzed using IBM SPSS for Windows version 25 (IBM Corp., Armonk, NY, USA). Categorical variables are presented as percentages and frequency distributions, whereas continuous variables are presented as the median and interquartile range (*IQR*). Kolmogorov-Smirnov and Shapiro-Wilk tests were used to determine whether the data were normally distributed. Because the continuous variables were non-normally distributed, they were compared using the Mann-Whitney U and Kruskal-Wallis tests, as appropriate. Categorical variables were compared using a chi-squared test or Fisher’s exact test, as appropriate. The correlation between the overall diagnostic confidence and accuracy was assessed using the Spearman’s correlation coefficient. Multivariable logistic regression analysis was used to identify the factors associated with having a diagnostic accuracy in the first quartile. The level of statistical significance was set at a *P* < 0.05.

## Results

### Participant characteristics

The survey was completed by 205 physicians (response rate = 34.2%), including 90 males (43.9%) and 115 females (56.1%). Overall, 90 participants (43.9%) had ≤1 year of experience, while 58 (28.3%) had at least 5 years of experience. In total, 89 physicians (43.4%) were medical interns, constituting the largest proportion of the study population, while 74 (36.1%) and 21 (10.2%) were family medicine and diagnostic radiology residents, respectively. Furthermore, 67 (32.7%) and 130 physicians (63.4%) reported having an interest in pulmonary medicine and diagnostic radiology, respectively. Greater than one half of the physicians (59.0%) had not undergone an elective rotation in diagnostic radiology. Additionally, 97 physicians (47.3%) reported having an adequate training in the interpretation of chest X-rays (Table 1).

**Table 1:** Characteristics of the participants

Variable	Male		Female		Total
	*N*	(%)	*N*	(%)	
Years in Practice					
≤ 1 year	36	(40.0)	54	(60.0)	90
2 years	10	(43.5)	13	(56.5)	23
3 years	7	(58.3)	5	(41.7)	12
4 years	7	(31.8)	15	(68.2)	22
≥ 5 years	30	(51.7)	28	(48.3)	58
Type of Training					
Medical Intern	36	(40.4)	53	(59.6)	89
General Practitioner	8	(38.1)	13	(61.9)	21
Family Medicine Resident	36	(48.6)	38	(51.4)	74
Radiology Resident	10	(47.6)	11	(52.4)	21
Interest in Pulmonary Medicine					
Yes	34	(50.7)	33	(49.3)	67
No	56	(40.6)	82	(59.4)	138
Interest in Diagnostic Radiology					
Yes	59	(45.4)	71	(54.6)	130
No	31	(41.3)	44	(58.7)	75
Had Elective in Diagnostic Radiology					
Yes	40	(47.6)	44	(52.4)	84
No	50	(41.3)	71	(58.7)	121
Had Adequate Training in Interpreting Chest X-rays					
Yes	38	(39.2)	59	(60.8)	97
No	52	(48.1)	56	(51.9)	108

*N* number of participants

### Diagnostic accuracy of interpretations

Table 2 summarizes the diagnostic accuracy and confidence of participants in interpreting chest X-ray images in the survey. The median number of cases that were answered correctly by the participants was 6 (*IQR*: 5–8), with an overall diagnostic accuracy of 63.1% (95% *CI*: 60.3–66.0%). Only 17 physicians (8.3%) interpreted all 10 cases correctly. Only five family medicine residents (6.8%) answered 9 (2.7%) or 10 (4.1%) of the cases correctly.

**Table 2:** Diagnostic accuracy and confidence in interpretation of the chest X-ray cases

Variable		Diagnostic Accuracy (%)	Diagnostic Confidence (%)
Clinical Case	Left Lower Lobe Pneumonia	51.8	63.6
	Normal Chest X-Ray	83.8	63.1
	Lung Abscess	68.0	70.5
	Pneumomediastinum	48.5	58.6
	Left Upper Lobe Atelectasis	49.4	61.5
	Pulmonary Edema	52.2	63.3
	Pneumoperitoneum	82.2	79.2
	Empyema	75.1	70.2
	Pneumothorax	73.4	77.8
	Coronavirus Disease 2019 Pneumonia	89.1	70.0
Training Type	Medical Interns	61.9	66.8
	General Practitioners	59.0	65.4
	Family Medicine Residents	58.0	63.9
	Diagnostic Radiology Residents	90.5	87.9
Overall		63.1	67.8

The cases of COVID-19 pneumonia and the pneumoperitoneum had the highest diagnostic accuracy scores, and were interpreted correctly by 175 (85.4%) and 165 physicians (80.5%), respectively. Less than one-half of participants interpreted the pneumomediastinum (42.0%), left lower lobe pneumonia (43.4%), and left upper lobe collapse cases (45.4%) correctly.

The diagnostic accuracy of family medicine residents (58.0%) was not significantly different from physicians who were not enrolled in postgraduate training programs, including medical interns and general practitioners (58.0% vs. 61.4%; *U* = 3649; *P* = 0.23); however, the diagnostic accuracy of family medicine residents was significantly less than that of the diagnostic radiology residents (58.0% vs. 90.5%; *U* = 118; *P* < 0.001). Physicians who had an interest or completed an elective rotation in diagnostic radiology achieved a higher diagnostic accuracy (*P* < 0.001).

Physicians who reported having adequate training in chest X-ray interpretation had a diagnostic accuracy of 68% compared to 59% in physicians who did not have adequate training (*U* = 4042; *P* = 0.004). Notably, family medicine residents reported the lowest rate (25.7%) of having an adequate training in chest X-ray interpretation (Table 3).

**Table 3:** Report of adequate training in interpreting chest X-rays according to different characteristics

Variable	Adequate Training in Interpreting Chest X-Ray				*P* value^a^
	Yes		No		
	N	(%)	N	(%)	
Gender					
Male	38	(42.2)	52	(57.8)	0.20
Female	59	(51.3)	56	(48.4)	
Years in Practice					
0–1 year	54	(60.0)	36	(40.0)	**0.01**
2 years	8	(34.8)	15	(65.2)	
3 years	7	(58.3)	5	(41.7)	
4 years	10	(45.5)	12	(54.5)	
≥ 5 years	18	(31.0)	40	(69.0)	
Type of Training					
Medical Intern	53	(59.6)	36	(40.4)	**< 0.01**
General Practitioner	7	(33.3)	14	(66.7)	
Family Medicine Resident	19	(25.7)	55	(74.3)	
Radiology Resident	18	(85.7)	3	(14.3)	
Diagnostic Accuracy	67.7%		59.0%		**< 0.01**

*N* number of participants

^a^*P* value in bold when significant

The diagnostic accuracy of family medicine residents who reported having adequate training in chest X-ray interpretation was slightly greater than family medicine residents who did not have adequate training in chest X-ray interpretation (65.3% vs. 55.5%; *U* = 358; *P* = 0.04). Interestingly, the diagnostic accuracy of family medicine residents did not differ significantly based on gender, years in practice, or having an interest in pulmonary medicine or diagnostic radiology (*P* > 0.05).

### Diagnostic confidence in interpretations

The overall diagnostic confidence in the interpretation of chest X-ray was 67.8%. There was a significant positive correlation (ρ = 0.39; *P* < 0.001) between the diagnostic confidence in the interpretation and the overall score attained by the physicians. Diagnostic radiology residents (87.9%) had the highest diagnostic confidence in interpreting the chest X-ray cases compared with medical interns (66.8%), general practitioners (65.4%), and family medicine residents (64.0%) (*P* < 0.001). Furthermore, physicians who reported having an adequate training in chest X-ray interpretation had a higher diagnostic confidence (72.6% vs. 63.4%; *U* = 3694; *P* < 0.001). However, the years of experience and the interest in diagnostic radiology or pulmonary medicine were not significantly associated with the diagnostic confidence (*P* > 0.05).

The physicians had the highest diagnostic confidence in the pneumoperitoneum (79.2%) and pneumothorax (77.8%) cases, whereas the physicians had the lowest diagnostic confidence in the pneumomediastinum (58.6%) and left upper lobe collapse cases (61.5%) (Table 2).

### Factors associated with diagnostic accuracy

The factors associated with diagnostic accuracy in the first quartile included being a diagnostic radiology resident (χ^2^ = 45.8; *P* < 0.001), having an interest in diagnostic radiology (χ^2^ = 8.2; *P* = 0.004), completing an elective rotation in diagnostic radiology (χ^2^ = 16.1; *P* < 0.001), having adequate chest X-ray training (χ^2^ = 6.3; *P* = 0.01), and overall diagnostic confidence in the interpretation (*U* = 6224; *P* < 0.001). Multivariable logistic regression analysis revealed that being a diagnostic radiology resident (*OR*: 13.0; 95% *CI*: 2.5–67.7) and having higher diagnostic confidence (*OR*: 2.2; 95% *CI*: 1.3–3.8) were the only independent predictors of achieving high diagnostic accuracy.

## Discussion

This study assessed the competency of family medicine residents in interpreting chest X-ray images of emergency conditions. There was a significant difference in the diagnostic accuracy between family medicine and diagnostic radiology residents. Furthermore, the competency of family medicine residents was comparable to physicians who had not undergone any postgraduate training, including medical interns and general practitioners.

It has been recognized that family medicine physicians often provide a lower quality of care at disease-specific levels with better outcomes for patients and lower costs than the other specialties. This phenomenon is called the “primary care paradox” and it has been well-described and investigated in the literature [13]. However, the clinical cases provided in the study were of common emergency conditions that all physicians should be able to recognize. It should be pointed out that the interpretation of chest X-ray by radiographers was found to be non-inferior to diagnostic radiologists [14].

**Fig. 1:**
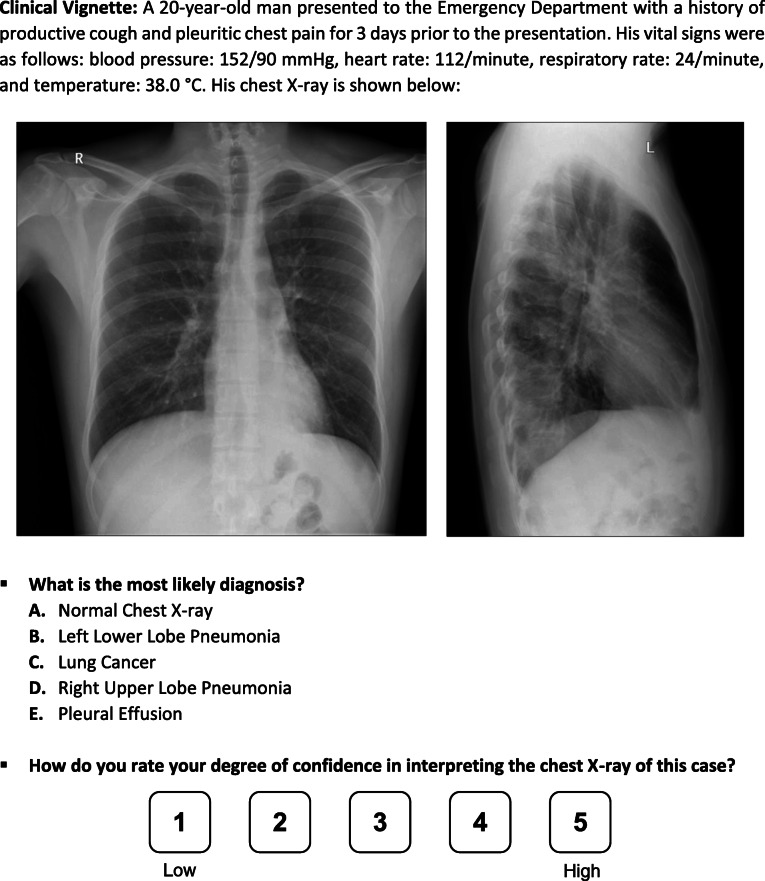
A question from the survey on a left lower lobe pneumonia case

Interestingly, the study showed that the lowest diagnostic accuracy of family medicine residents was in interpreting the case of left lower lobe pneumonia. It should be noted that pneumonia is the most common indication for obtaining a chest X-ray in general practice [4]. A chest X-ray is a valuable tool in the assessment of patients with suspected pneumonia because the clinical presentation alone is not predictive of pneumonia and a chest X-ray substantially reduce the number of misdiagnoses [15]. The low diagnostic accuracy for the interpretation of left lower lobe pneumonia in the present study could be attributable to the subtle abnormality on the frontal chest X-ray; however, the consolidation was clearly evident on the lateral view (Fig. 1). A similar study by Satia et al. [16] showed that lower lobe atelectasis is the least correctly interpreted chest X-ray. Indeed, lower lobe pathology is better evaluated by a lateral view chest X-ray [17].

**Fig. 2:**
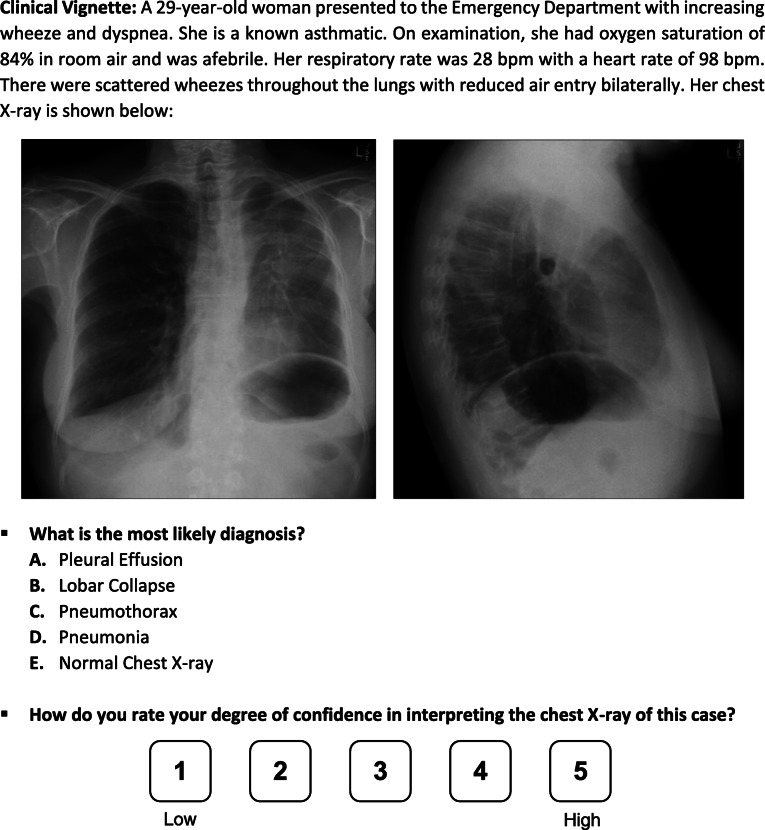
A question from the survey on a left upper lobe collapse case

Despite its importance, the lateral view is often ignored during routine chest X-ray evaluation. The lateral view is useful in interpreting findings that are not clearly shown on the frontal view because 15% of the lung is obscured by cardiovascular and mediastinal structures [17, 18]. Hence, the lateral view is of paramount importance to visualize the hidden areas of the lung in the frontal view. The low diagnostic accuracy of family medicine residents in recognizing pneumonia in the present study cannot be generalized to all cases of pneumonia considering the wide spectrum of radiological manifestations of pneumonia [19]. Similarly, lobar atelectasis has different radiological findings based on the anatomic location of the involved lobe; however, left upper lobe collapse has a unique manifestation as the lobe collapses anteriorly giving a characteristic veiling opacity (Fig. 2).

**Fig. 3:**
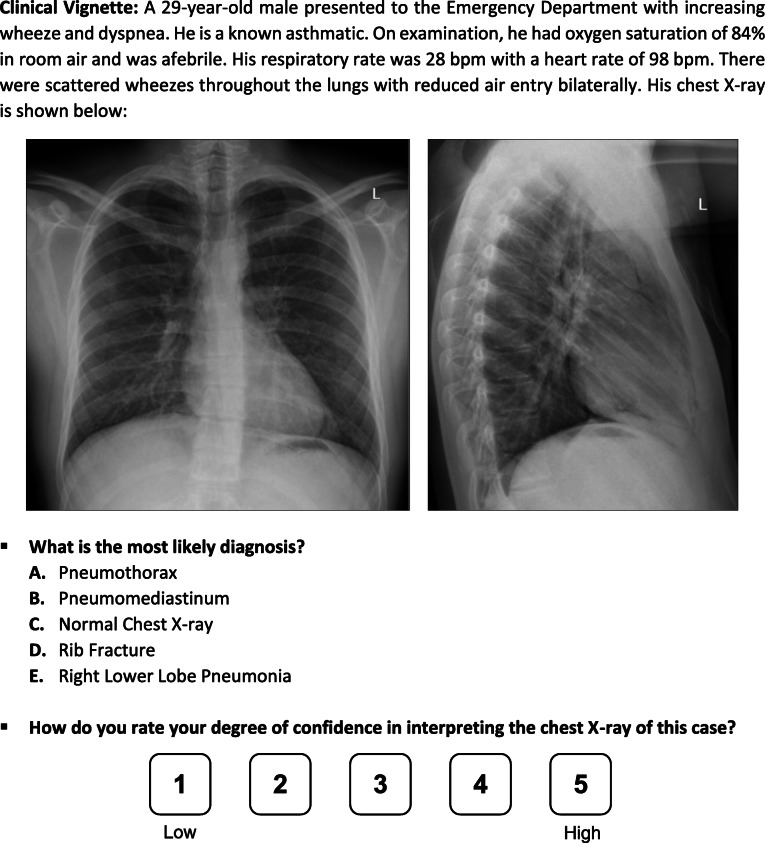
A question from the survey on a pneumomediastinum case

The pneumomediastinum case was interpreted correctly by less than one-half of the participants, and was the case interpreted correctly least often. This finding is consistent with a previous study that estimated 50% of pneumomediastinum cases are missed on frontal chest X-rays [20]. Several imaging findings of thoracic conditions have been linked to radiological signs and symbols. The knowledge of these associations is helpful, for both radiologists and non-radiologists, and could aid in the interpretation of chest X-ray images [21]. For example, a pneumomediastinum may manifest on frontal chest X-ray as a radiolucency between the heart and the superior surface of the diaphragm. Such a finding is referred to as a “continuous diaphragmatic sign” (Fig. 3) [22].

**Fig. 4:**
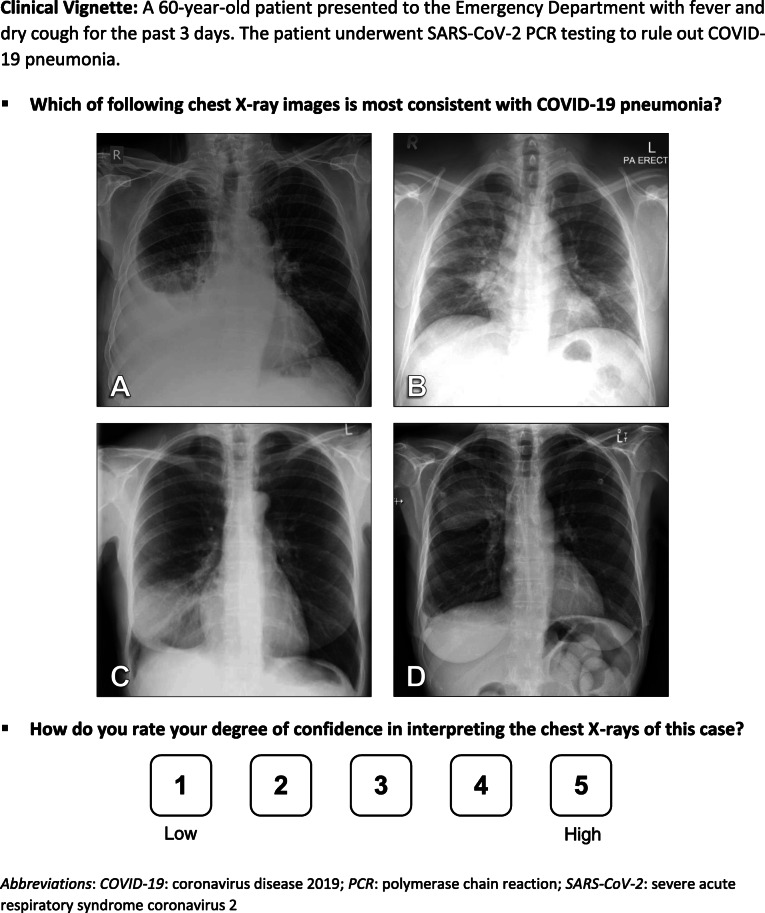
A question from the survey on a COVID-19 pneumonia case

The COVID-19 pneumonia case (Fig. 4) was correctly answered by the highest number of participants. Considering that the study was conducted in the midst of the COVID-19 pandemic, this finding might not be surprising. Prior exposure to similar radiological images has been shown to be associated with an increased likelihood of accurate and correct interpretations [23]. This finding stressed the importance of having adequate exposure for family medicine residents to medical images to enhance their diagnostic abilities.

Notably, one-fifth of the participants did not interpret the normal chest X-ray correctly. Misdiagnoses of normal chest X-rays may subject patients to inappropriate management with potential adverse effects and complications. While the chest X-ray used in this study was entirely normal, it should be noted there can be significant differences in interpretation of parenchymal findings on chest X-rays regardless of the experience of the physician [24]. Furthermore, a previous study showed that 30% of patients who were assumed to have community-acquired pneumonia based on clinical and chest X-ray findings were found to have no evidence of pneumonia on computed tomography scans [25].

The current study showed a significant positive correlation between the diagnostic confidence and accuracy in chest X-ray interpretation. We assume that when a physician is confident in the interpretation of a chest X-ray, the interpretation is more likely to be accurate. Diagnostic radiology residents were shown to have the highest diagnostic confidence and accuracy; however, the diagnostic confidence was evaluated using a subjective 5-point scale, thus making the comparison among physicians of limited value. The current study did not demonstrate an association between diagnostic confidence or diagnostic accuracy and years in practice. Surprisingly, Satia et al. [16] reported that the diagnostic accuracy of specialist registrars was higher than non-respiratory medicine consultants. It was shown that the self-assessment of physicians is of limited accuracy and external assessment, as such in this study, is needed to correctly assess the competency and skills of physicians [26].

The current study findings demonstrated the need for interventions targeting family medicine residents to improve chest X-ray interpretation skills. Family medicine residents who reported having adequate training in chest X-ray interpretation had higher diagnostic accuracy and confidence. There is a pressing need to implement a radiology training course in the family medicine residency program. Today, radiology courses could be delivered in a wide variety of formats, including an online platform [27]. Several participants welcomed the idea of this survey and requested further similar online quizzes. Furthermore, introducing a rotation in the diagnostic radiology department during the family medicine residency program should be considered.

A particular feedback comment caught our attention: “the survey has a good selection of cases; however, the provision of clinical vignettes made the interpretation easier.” This comment is valid because previous studies showed that diagnostic accuracy and confidence increase when clinical information is provided [28, 29]. While the clinical vignettes we provided were not indicative of a specific diagnosis, the interpretation of a chest X-ray should not be made in isolation from the clinical information. For example, a patient with a pleural effusion will be diagnosed with a hemothorax when a clinical history of trauma is provided. Similarly, a chest X-ray with bilateral pulmonary infiltrates might be diagnosed as pulmonary edema. If a clinical history of fever and productive cough is provided, however, it is more likely to be diagnosed as multifocal pneumonia. Hence, providing clinical information is of paramount importance in chest X-ray interpretation. The clinical information, however, may be a distraction and lead to false-positive interpretations. Therefore, it is suggested to review the radiological images before reading the clinical data [30].

This is the first study which investigated the competency of family medicine residents in the Saudi Board of Family Medicine Program in interpreting chest X-rays for emergency conditions in comparison with diagnostic radiology residents. Several participants acknowledged the invitation for the survey and appreciated the option to receive the correct answers after the study was completed. We believe that this motivator along with the reminder e-mails helped to achieve a reasonable response rate because the use of incentive strategies has been shown to improve the response rates in online surveys [12].

The present study had some limitations. The survey-based methodology could have introduced some bias. Physicians who had a greater interest in chest X-ray interpretation might have been more likely to respond. Furthermore, the study used a limited number of chest X-rays to assess the interpretation skills. For example, skeletal radiographs and pediatric chest X-rays were not included. The radiological appearances of the pathologies on chest X-rays in the present study were classic, which might not be the case in real-life medicine. Lastly, the majority of participants completed the survey using their mobile phones, which could be less optimal than reading the images on computer monitors, as reported by one of the participants.

## Conclusion

The competency of family medicine residents in the interpretation of chest X-rays for emergency conditions is far from optimal. A radiology training course focusing on the acute and life-threatening conditions seems a good starting point if the family medicine residents are expected to make clinical decisions based on chest X-ray interpretations. Alternatively, the use of tele-radiology in primary care could be considered. Further research is needed to re-assess the interpretation skills of family medicine residents after implementation of improvement measures.

## Supplementary Information

**Additional file 1.** The chest X-ray survey questionnaire.

## Data Availability

The datasets used and/or analyzed during the current study are available from the corresponding author on reasonable request.

## Abbreviations

CI: Confidence Interval
COVID-19: Coronavirus Disease 2019
IQR: Interquartile Range
OR: Odds Ratio
