# Supplementary material for: Family medicine residents’ skill levels in emergency chest X-ray interpretation
Source: BMC Fam Pract. 2021 Feb 17;22:39. doi: 10.1186/s12875-021-01390-3 (PMC7889059; doi:10.1186/s12875-021-01390-3)
Supplement: Supplementary file 1 — Additional file 1. The chest X-ray survey questionnaire. [file 12875_2021_1390_MOESM1_ESM.docx]

**Demographic Information**

- **What is your gender?**
- Male
- Female
- **How long have you been in clinical practice?**
- ≤ 1 year
- 2 years
- 3 years
- 4 years
- ≥ 5 years
- **What is your current training level?**
- Medical Intern
- General Practitioner
- Family Medicine Resident
- Diagnostic Radiology Resident
- **What is your current training level?**
- Medical Intern
- General Practitioner
- Family Medicine Resident
- Diagnostic Radiology Resident
- **Do you have interest in pulmonary medicine?**
- Yes
- No
- **Do you have interest in diagnostic radiology?**
- Yes
- No
- **Have you had an elective rotation in diagnostic radiology?**
- Yes
- No
- **Have you had an adequate training in chest X-ray interpretation?**
- Yes
- No

**Question 1**

**Clinical Vignette:** A 20-year-old man presented to the Emergency Department with a history of productive cough and pleuritic chest pain for 3 days prior to the presentation. His vital signs were as follows: blood pressure: 152/90 mmHg, heart rate: 112/minute, respiratory rate: 24/minute, and temperature: 38.0 °C. His chest X-ray is shown below:


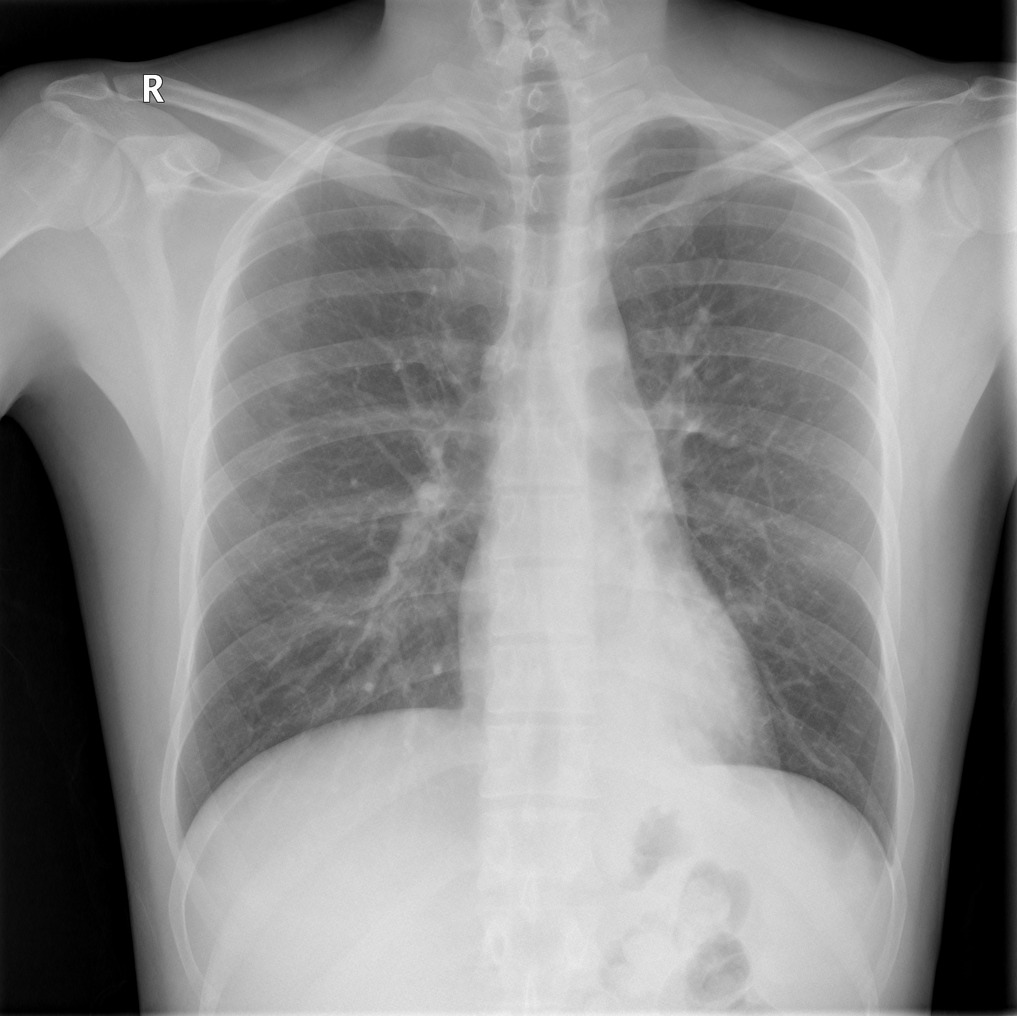

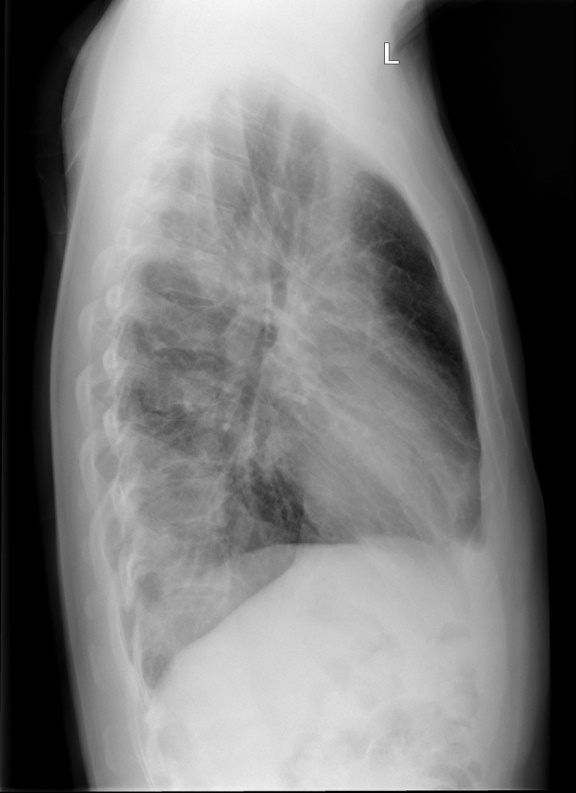


- **What is the most likely diagnosis?**

1. Normal Chest X-ray
2. Left Lower Lobe Pneumonia
3. Lung Cancer
4. Right Upper Lobe Pneumonia
5. Pleural Effusion

- **How do you rate your degree of confidence in interpreting the chest X-ray of this case?**

**1**

**2**

**3**

**4**

**5**

Low

High

**Question 2**

**Clinical Vignette:** A 50-year-old male patient developed a dry cough for 3 days prior to his presentation to the Emergency Department. He is a heavy smoker. His vital signs were as follows: Blood Pressure: 152/90 mmHg, Heart Rate: 100 bpm, Respiratory Rate: 16 bpm, and Temperature: 37.0 C:


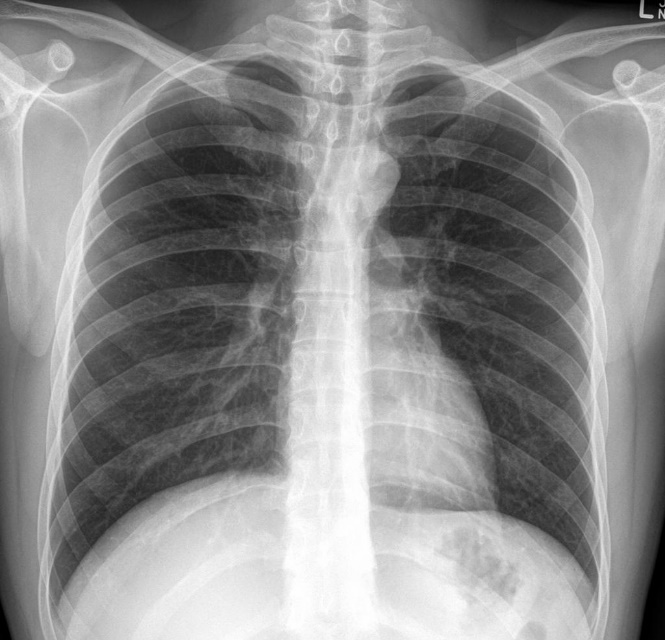


- **What is the most likely diagnosis?**

1. Normal Chest X-Ray
2. Pneumonia (Right Upper Lobe)
3. Pneumonia (Right Lower Lobe)
4. Pleural Effusion
5. Lung Cancer

- **How do you rate your degree of confidence in interpreting the chest X-ray of this case?**

**1**

**2**

**3**

**4**

**5**

Low

High

**Question 3**

**Clinical Vignette:** A 67-year-old male presents to the Emergency Department with a productive cough, nausea and vomiting. He had a previous stroke. He is a non-smoker. On examination, he has saturation of 85% in air, has a HR of 100 bpm, and is febrile with a temperature of 39.5°C.


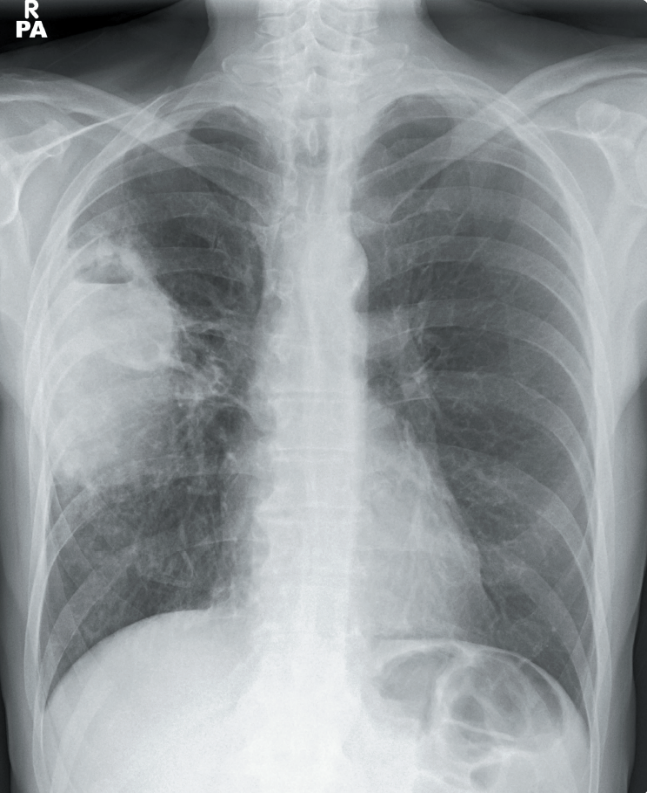


- **What is the most likely diagnosis?**

1. Right Middle Lobe Pneumonia
2. Right Upper Lobe Pneumonia
3. Tuberculosis
4. Lung Abscess
5. Lung Cancer

- **How do you rate your degree of confidence in interpreting the chest X-ray of this case?**

**1**

**2**

**3**

**4**

**5**

Low

High

**Question 4**

**Clinical Vignette:** A 29-year-old male presented to the Emergency Department with increasing wheeze and dyspnea. He is a known asthmatic. On examination, he had oxygen saturation of 84% in room air and was afebrile. His respiratory rate was 28 bpm with a heart rate of 98 bpm. There were scattered wheezes throughout the lungs with reduced air entry bilaterally. His chest X-ray is shown below:


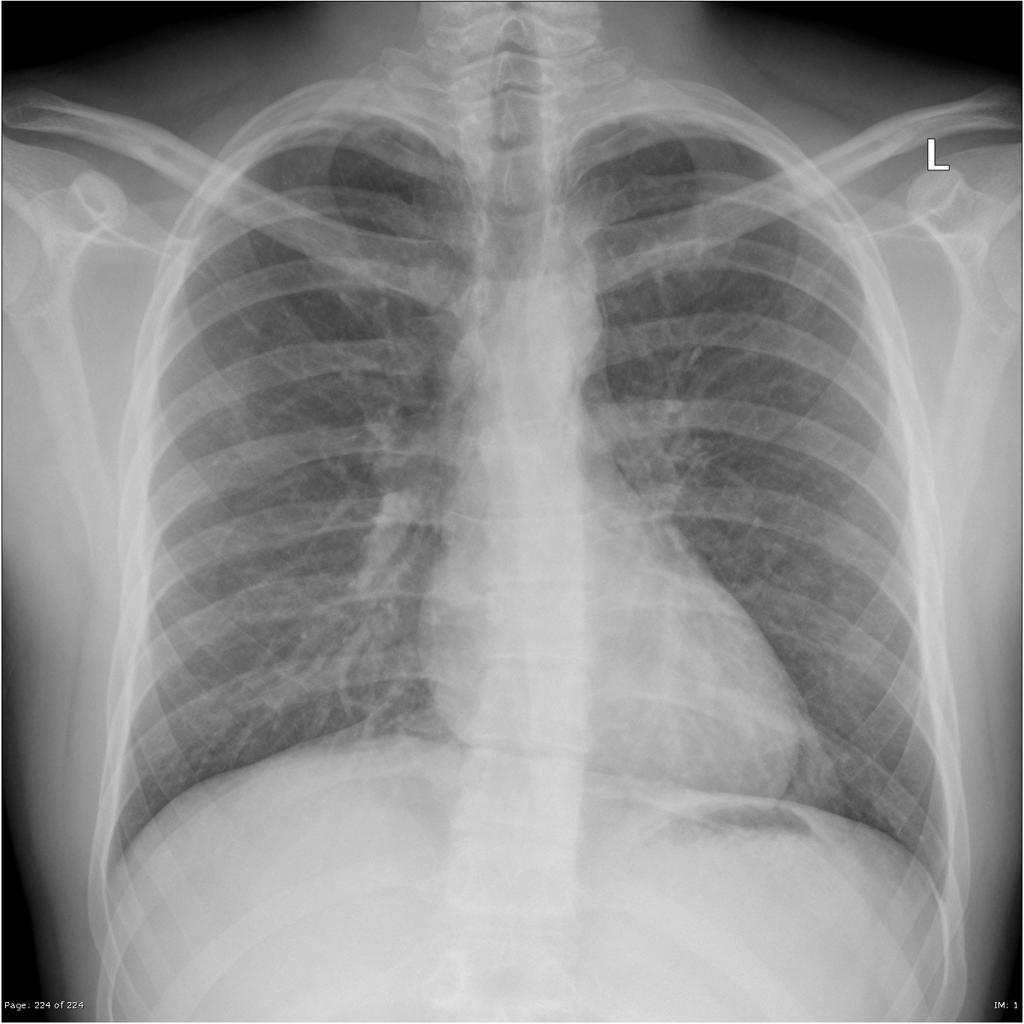

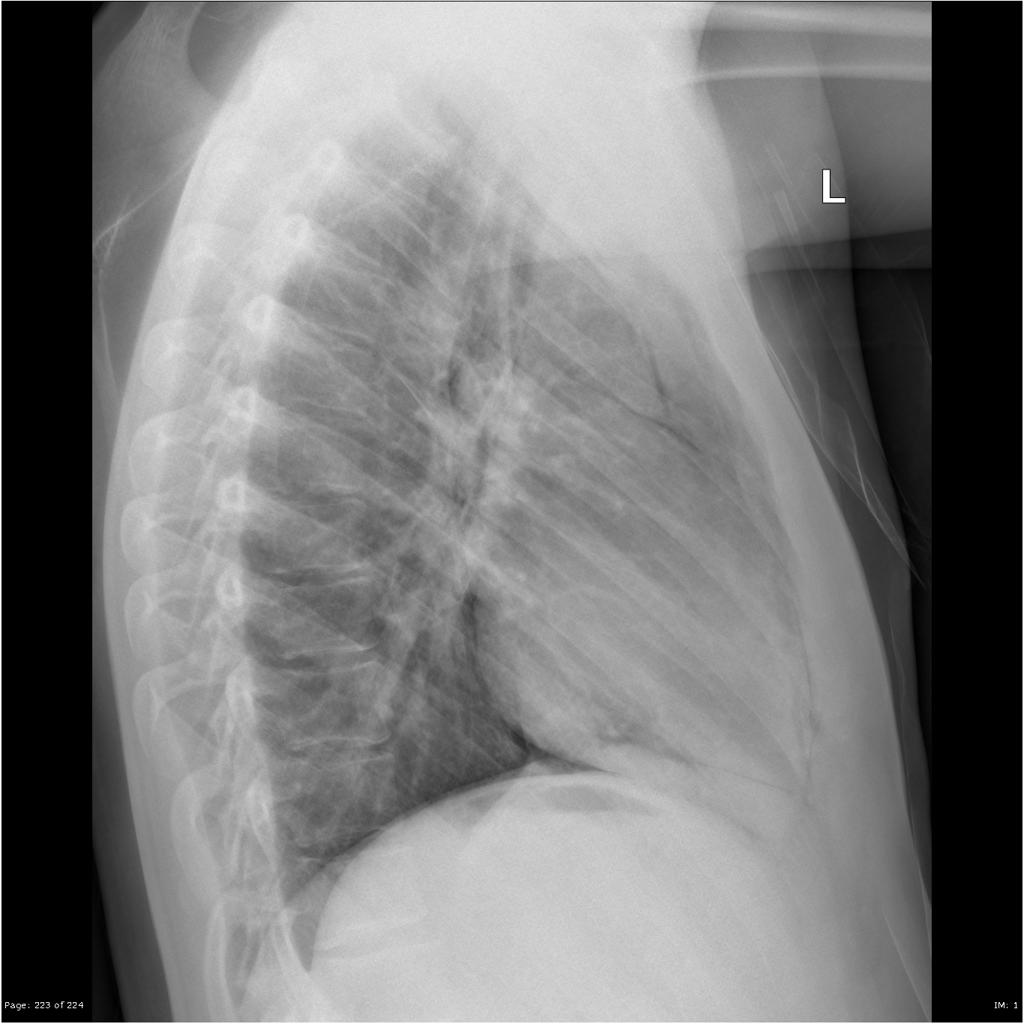


- **What is the most likely diagnosis?**

1. Pneumothorax
2. Pneumomediastinum
3. Normal Chest X-ray
4. Rib Fracture
5. Right Lower Lobe Pneumonia

- **How do you rate your degree of confidence in interpreting the chest X-ray of this case?**

**1**

**2**

**3**

**4**

**5**

Low

High

**Question 5**

**Clinical Vignette:** A 29-year-old woman presented to the Emergency Department with increasing wheeze and dyspnea. She is a known asthmatic. On examination, she had oxygen saturation of 84% in room air and was afebrile. Her respiratory rate was 28 bpm with a heart rate of 98 bpm. There were scattered wheezes throughout the lungs with reduced air entry bilaterally. Her chest X-ray is shown below:


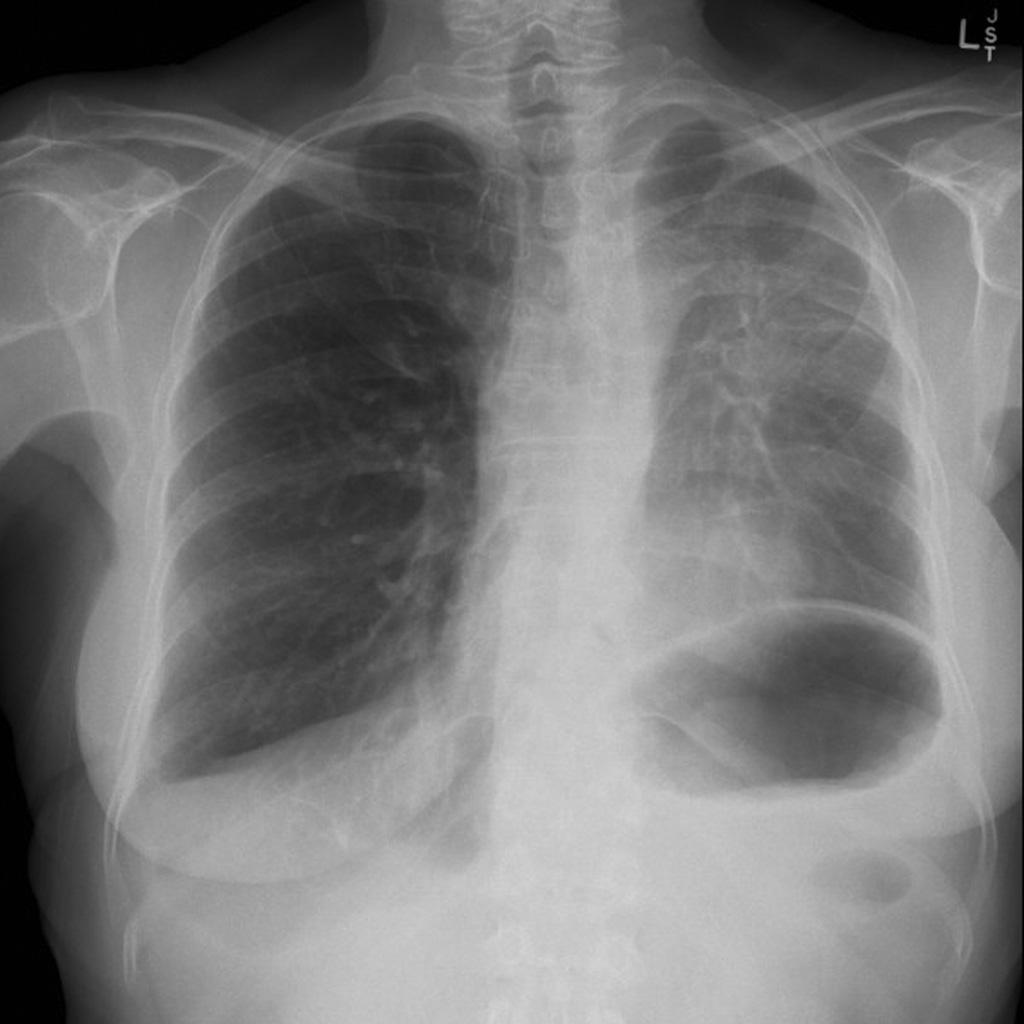

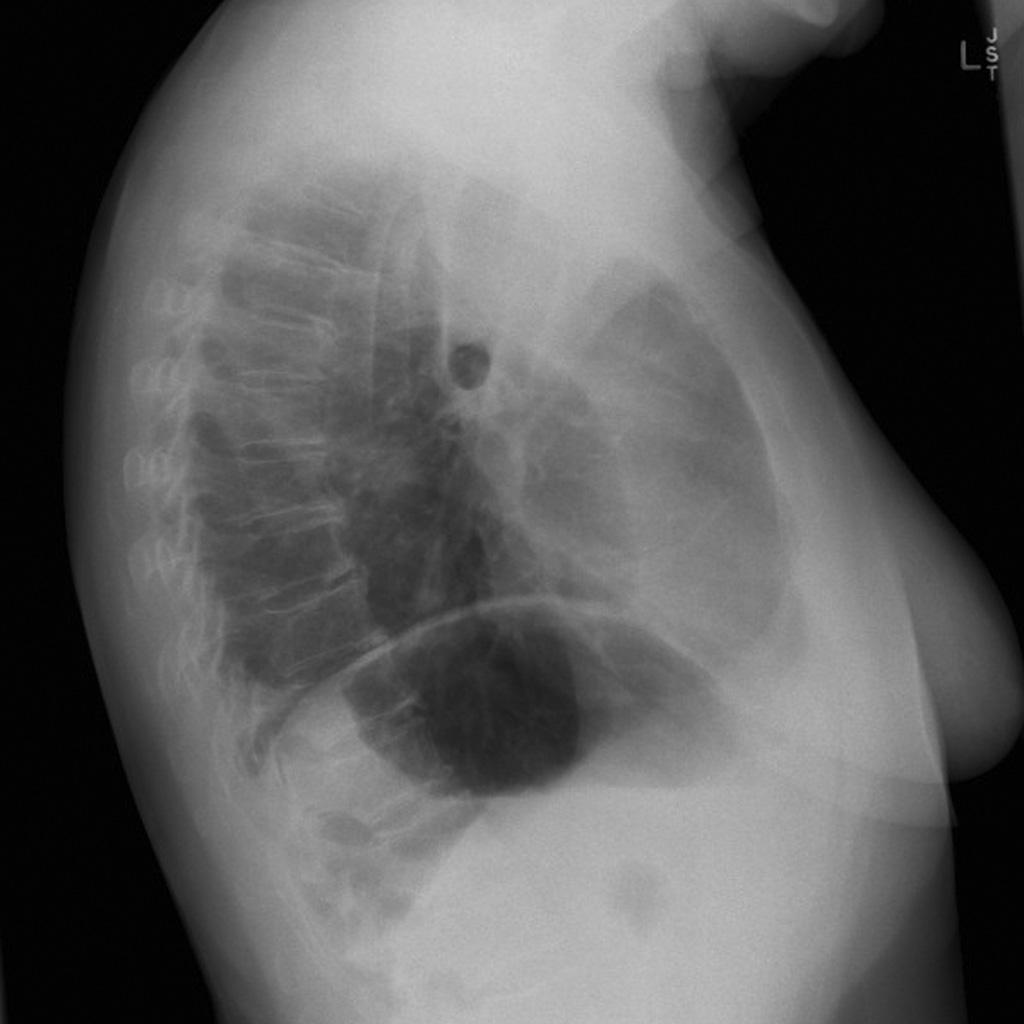


- **What is the most likely diagnosis?**

1. Pleural Effusion
2. Lobar Collapse
3. Pneumothorax
4. Pneumonia
5. Normal Chest X-ray

- **How do you rate your degree of confidence in interpreting the chest X-ray of this case?**

**1**

**2**

**3**

**4**

**5**

Low

High

**Question 6**

**Clinical Vignette:** A 60-year-old man presents to the Emergency Department with acute shortness of breath. He has a 20 pack-year smoking history. On examination, he is afebrile with saturations of 90% in air. His heart rate is 100 bpm with a respiratory rate of 22. There is dullness and inspiratory crackles in both lower zones. His chest X-ray is shown below:


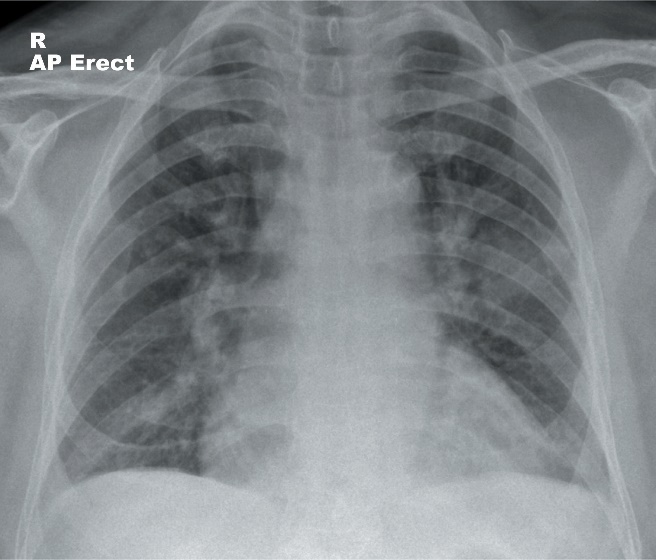


- **What is the most likely diagnosis?**

1. Pneumonia
2. Pulmonary Edema
3. Interstitial Lung Disease
4. Aortic Dissection
5. Normal Chest X-ray

- **How do you rate your degree of confidence in interpreting the chest X-ray of this case?**

**1**

**2**

**3**

**4**

**5**

Low

High

**Question 7**

**Clinical Vignette:** A 25-year-old male presents to the Emergency Department with worsening abdominal pain. He is a non-smoker. On examination, he has an oxygen saturation of 87% in air, respiratory rate of 22 bpm, heart rate of 130 bpm, and is febrile with a temperature of 37.9°C. His chest X-ray is shown below:

**
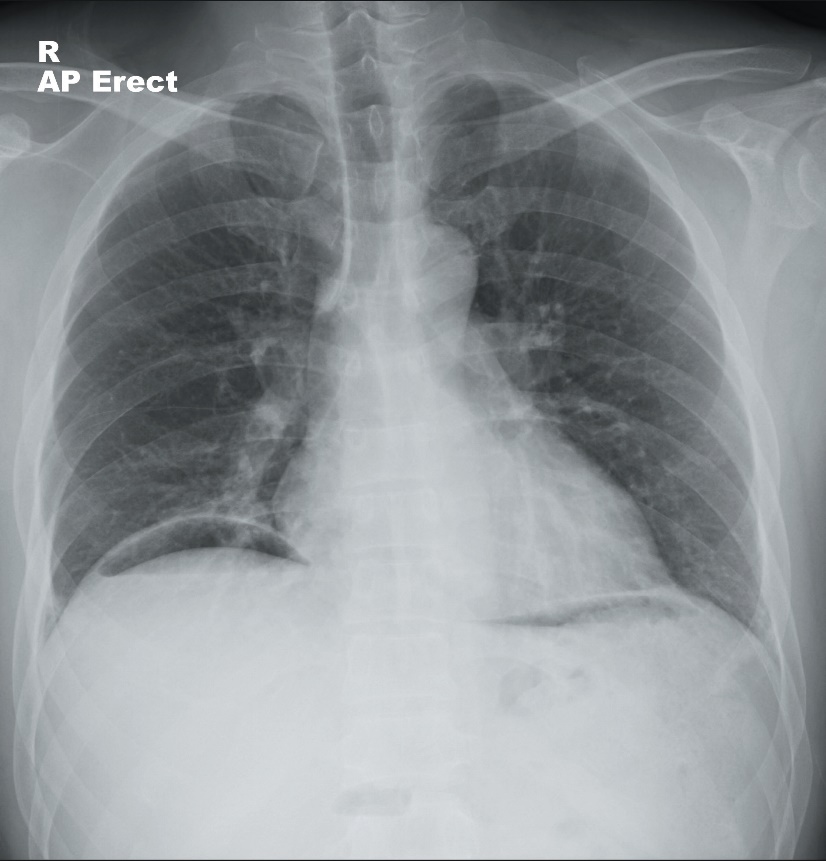
**

- **What is the most likely diagnosis?**

1. Hiatal Hernia
2. Pleural Effusion
3. Pneumothorax
4. Pneumoperitoneum
5. Pneumonia

- **How do you rate your degree of confidence in interpreting the chest X-ray of this case?**

**1**

**2**

**3**

**4**

**5**

Low

High

**Question 8**

**Clinical Vignette:** A 70-year-old male presents to the Emergency Department feeling unwell with a productive cough. He was diagnosed with pneumonia 3 weeks earlier, but has not been taking his antibiotics. He is a heavy smoker. On examination, he is febrile and has a saturation of 90% in air. There are crackles, reduced air entry in the right hemithorax. His chest X-ray is shown below:


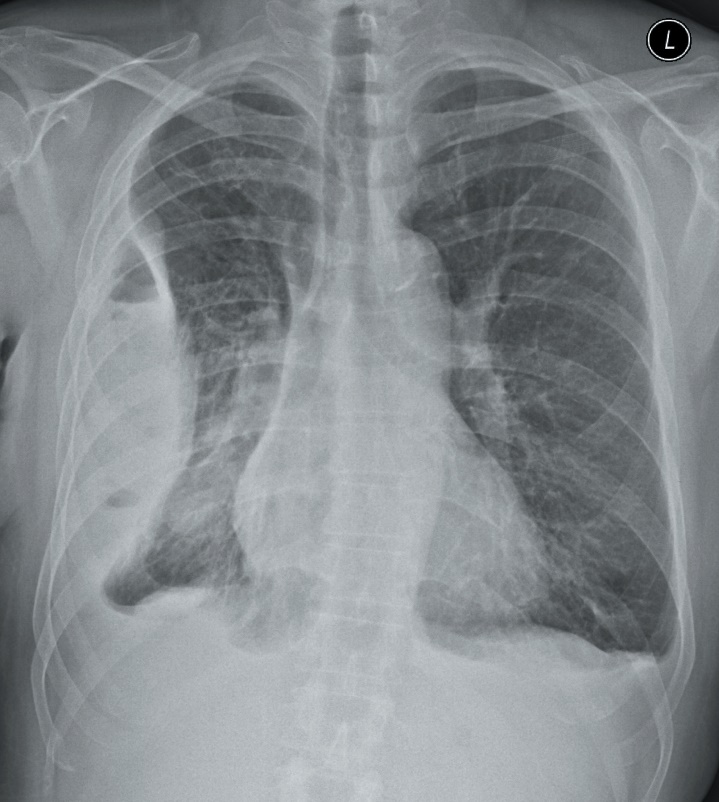


- **What is the most likely diagnosis?**

1. Lobar Collapse
2. Tuberculosis
3. Pneumothorax
4. Empyema
5. Lung Abscess

- **How do you rate your degree of confidence in interpreting the chest X-ray of this case?**

**1**

**2**

**3**

**4**

**5**

Low

High

**Question 9**

**Clinical Vignette:** A 20-year-old male presents to the Emergency Department with sudden onset right-sided pleuritic chest pain and breathlessness. He has no significant past medical history and is a non-smoker. On examination, he has an oxygen saturation of 93% in air and is afebrile. His heart rate is 95bpm, and blood pressure is 120/82 mmHg. His chest X-ray is shown below:


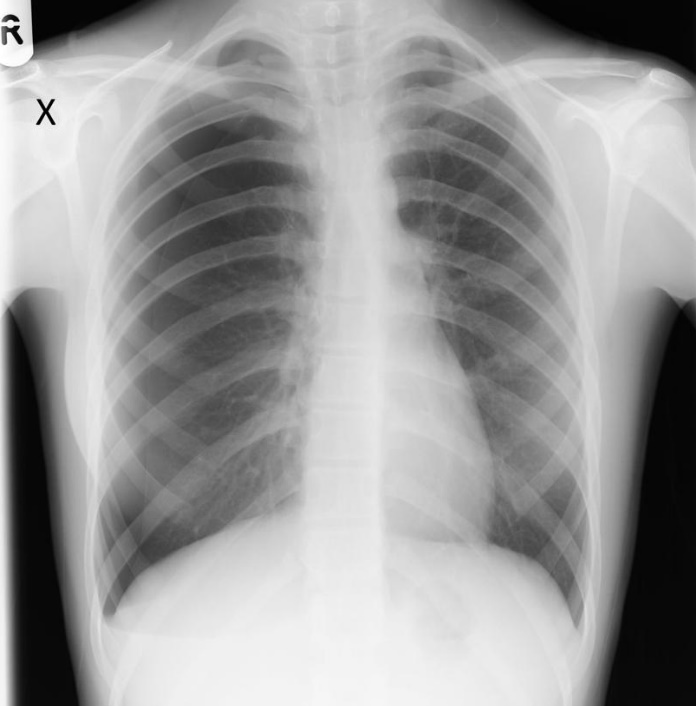


- **What is the most likely diagnosis?**

1. Normal Chest X-ray
2. Pneumonia
3. Pneumothorax
4. Pleural Effusion
5. Rib Fracture

- **How do you rate your degree of confidence in interpreting the chest X-ray of this case?**

**1**

**2**

**3**

**4**

**5**

Low

High

**Question 10**

**Clinical Vignette:** A 60-year-old patient presented to the Emergency Department with fever and dry cough for the past 3 days. The patient underwent SARS-CoV-2 PCR testing to rule out COVID-19 pneumonia.

- **Which of following chest X-ray images is most consistent with COVID-19 pneumonia?**

**B**

**C**

**D**

**A**


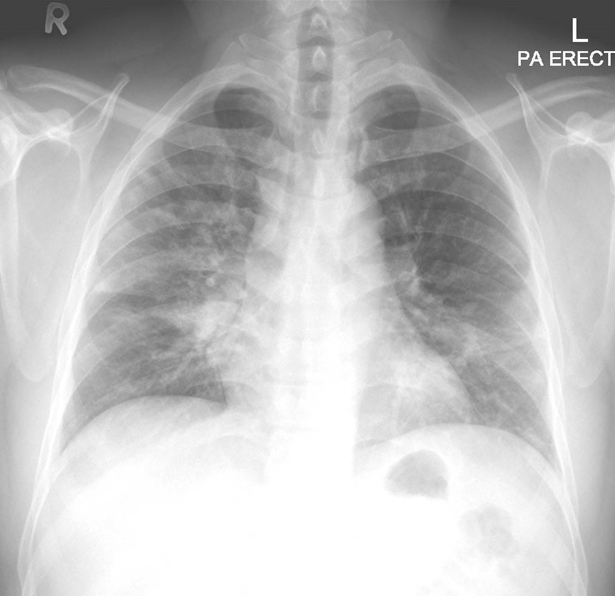

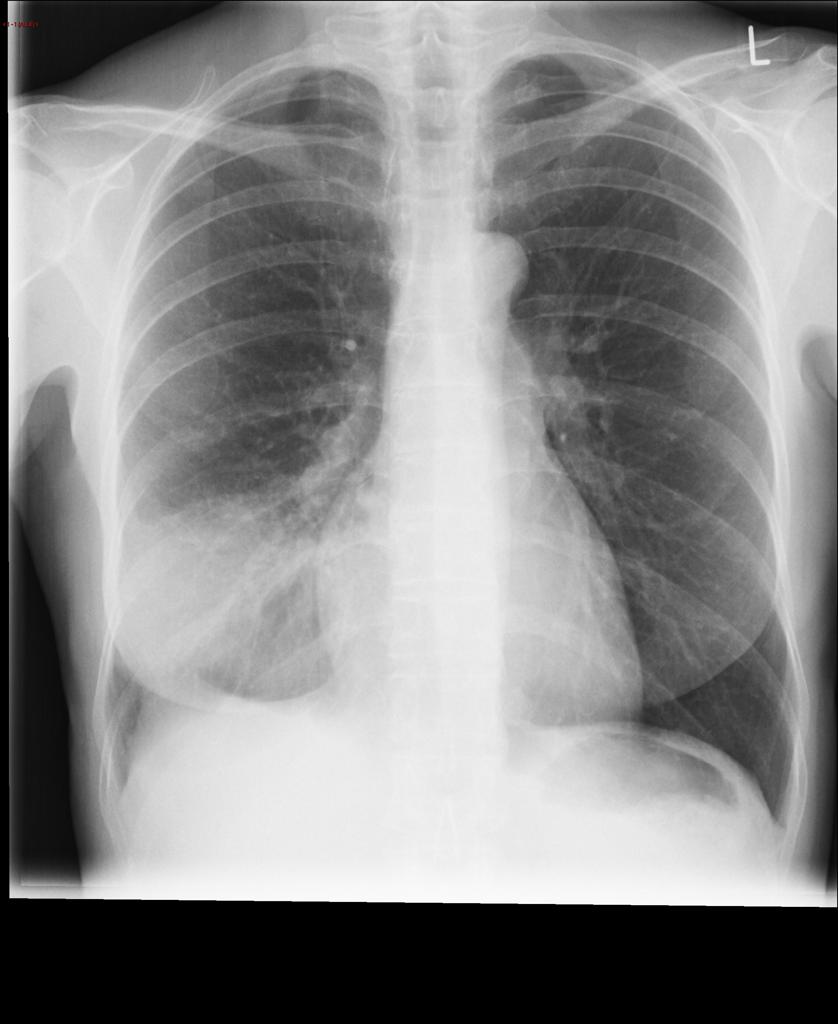

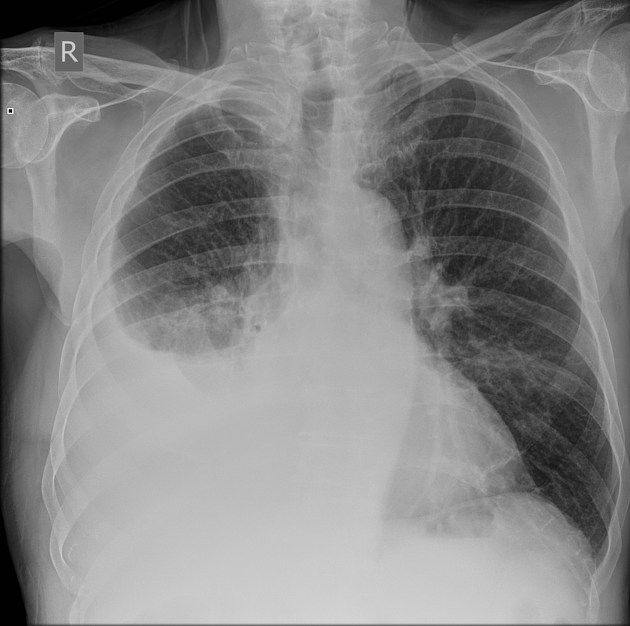

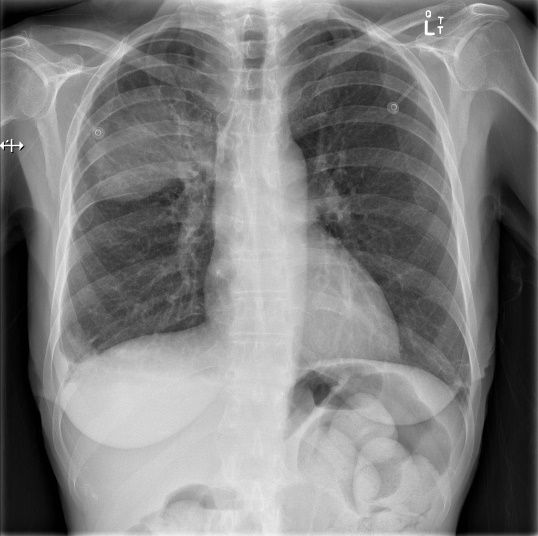


- **How do you rate your degree of confidence in interpreting the chest X-rays of this case?**

**1**

**2**

**3**

**4**

**5**

Low

High
